# Assessment of trochanteric vascularity using quantitative magnetic resonance imaging in a cadaveric model

**DOI:** 10.1002/jeo2.70092

**Published:** 2024-11-14

**Authors:** Craig E. Klinger, Burak Altintas, Kathryn A. Barth, Kenneth M. Lin, David C. Dewar, Lionel E. Lazaro, Jonathan P. Dyke, David S. Wellman, David L. Helfet

**Affiliations:** ^1^ Orthopaedic Trauma Service, Hospital for Special Surgery, Weill Cornell Medicine New York New York USA; ^2^ Department of Orthopaedic Surgery Stanford University Stanford California USA; ^3^ Department of Orthopaedic Surgery University of Newcastle Newcastle New South Wales Australia; ^4^ Doctors' Center Hospital San Juan and Sabanera Health Dorado Dorado Puerto Rico USA; ^5^ Citigroup Biomedical Imaging Center, Weill Cornell Medicine New York New York USA; ^6^ Department of Orthopaedic Surgery Westchester Medical Center, New York Medical College New York New York USA

**Keywords:** computed tomography, lateral femoral circumflex artery, medial femoral circumflex artery, quantitative MRI, trochanteric vascularity

## Abstract

**Purpose:**

Few studies have assessed trochanteric vascularity despite its implications for bone healing and surgical approaches. This study aimed to assess the regional arterial contributions of the medial femoral circumflex artery (MFCA) versus the lateral femoral circumflex artery (LFCA) to trochanteric vascularity.

**Methods:**

Ten adult human cadaveric pelvises to mid‐femur specimens were obtained. One hip was randomly assigned experimental (either MFCA or LFCA MRI‐contrast infusion) and contralateral as control (MFCA and LFCA magnetic resonance imaging [MRI]‐contrast infusion). Vascular dissection was performed for MFCA and LFCA cannulation. Pre‐ and post‐contrast 3T MRI was completed, and intra‐osseous contributions were quantified by region: greater trochanter (GT), intertrochanteric (IT), lesser trochanter (LT) and subtrochanteric (ST). A polyurethane compound mixed with barium sulfate was injected into the LFCA cannula, and into the MFCA cannula for the contralateral hip. Computed tomography (CT) imaging was completed to assess terminal branch locations.

**Results:**

MFCA provided the majority of arterial contributions to the full trochanteric region (68.5% MFCA, 31.5% LFCA; standard deviation [SD]: 10.7%, *p* < 0.001). Over 70% of arterial contributions to ST, LT and IT regions are derived from MFCA. GT contributions were more balanced (52.5% MFCA, 47.5% LFCA; SD: 33.7%; *p* = 0.853). Significant differences were found between MFCA and LFCA contributions in all regions except for the GT. CT revealed multiple consistent MFCA and LFCA trochanteric terminal branches.

**Conclusions:**

MFCA provided the dominant trochanteric arterial supply, which highlights MFCA's importance to overall hip vascularity. LFCA's trochanteric contribution was smaller but still provided trochanteric contributions, especially the GT region. Knowledge of trochanteric arterial contributions can be beneficial for optimizing surgical approaches and fixation to protect terminal branches during trochanteric fracture, nonunion treatment and trochanteric osteotomies.

**Level of Evidence:**

Not applicable.

**Abbreviations**
CT: computed tomography
Gd‐DTPA: gadolinium‐diethylenetriamine pentaacetic acid
GT: greater trochanter
IT: intertrochanteric
LFCA: lateral femoral circumflex artery
LT: lesser trochanter
MFCA: medial femoral circumflex artery
SD: standard deviation
ST: subtrochanteric

## BACKGROUND

Fractures involving the trochanteric region are common, accounting for a large proportion of proximal femoral fractures [8, 22]. Additionally, trochanteric osteotomies are often utilized for surgical approaches and exposures in trauma surgery, hip preservation, and arthroplasty. While modern fixation constructs result in high union rates for trochanteric fractures, nonunion remains a concern due to the high overall prevalence of these fractures [4]. The fracture pattern, surgical technique, and bone quality contribute to nonunion risk [1, 2, 3, 4, 5, 7, 8, 12, 13, 19, 22, 25, 27, 28, 29, 30, 31]. Careful consideration of the blood supply during surgical procedures can help reduce the risk of nonunion. Moreover, preservation of the blood supply is of particular concern in the setting of osteotomies, for which nonunion is of increased concern [6]. Nonunion of the greater trochanter can result in abductor dysfunction and subsequent pain, limp, and impairment.

The vascular supply to the femoral head and neck has been well described both qualitatively and quantitatively, with the dominant arterial supply provided by the deep branch of the medial femoral circumflex artery (MFCA) [10, 14, 16, 17, 18, 19, 28, 29, 30]. Prior research by Grose et al. using latex injection in a cadaveric model also found anastomoses in seven of eight specimens between the inferior gluteal artery and MFCA adjacent to the tendon of obturator externus [11].

Angiography following hip surgery suggests a decrease in arterial perfusion to the greater trochanteric region following ligation of the lateral femoral circumflex artery (LFCA), suggesting the LFCA may also play a significant role in the blood supply to this region of the proximal femur [15]. To our knowledge, no studies have characterized the vascular supply to the trochanteric region. Quantitative magnetic resonance imaging (MRI) is a well‐established technique that defines relative arterial contributions to defined regions of interest [7, 9, 20, 21].

Therefore, the aim of this study was to assess and compare the arterial contributions of the MFCA versus LFCA to regional trochanteric vascularity using quantitative MRI. We hypothesized that the MFCA would provide the dominant arterial supply to the trochanteric region.

## METHODS

After approval was obtained by our institutional review board for this cadaveric research study, 10 cadaveric human fresh‐frozen pelvic specimens were obtained (including pelvis with bilateral hips to mid‐femur) from Anatomy Gifts Registry. This study was a sub‐analysis of a prior study by our group which assessed the relative contribution of the MFCA and LFCA to the regional vascularity of the head and neck of the femur also using a quantitative MRI protocol and the same cadaveric specimens [7]. The specimens included had a mean age of 54.3 years (standard deviation [SD]: 13.5, range: 28–69) and mean BMI of 23.1 (SD: 8.0, range: 13–36). The group of specimens was composed of seven males and three females. Any specimen with a known history of vascular disease (including diabetes mellitus), prior hip trauma or prior hip surgery was not acquired for this study and was excluded at the time of screening. The cadaveric specimens acquired and utilized for this study had the following causes of death: (2) cancer, (2) pneumonia, (1) drug overdose, (1) achalasia‐related complications, (1) acute respiratory failure, (1) anoxic brain injury, (1) cardiac arrest, (1) unspecified. All surgical procedures in this study were completed within our institutional anatomy laboratory and advanced imaging was acquired at our research radiographic imaging center affiliate.

### MRI protocol

**Figure 1:**
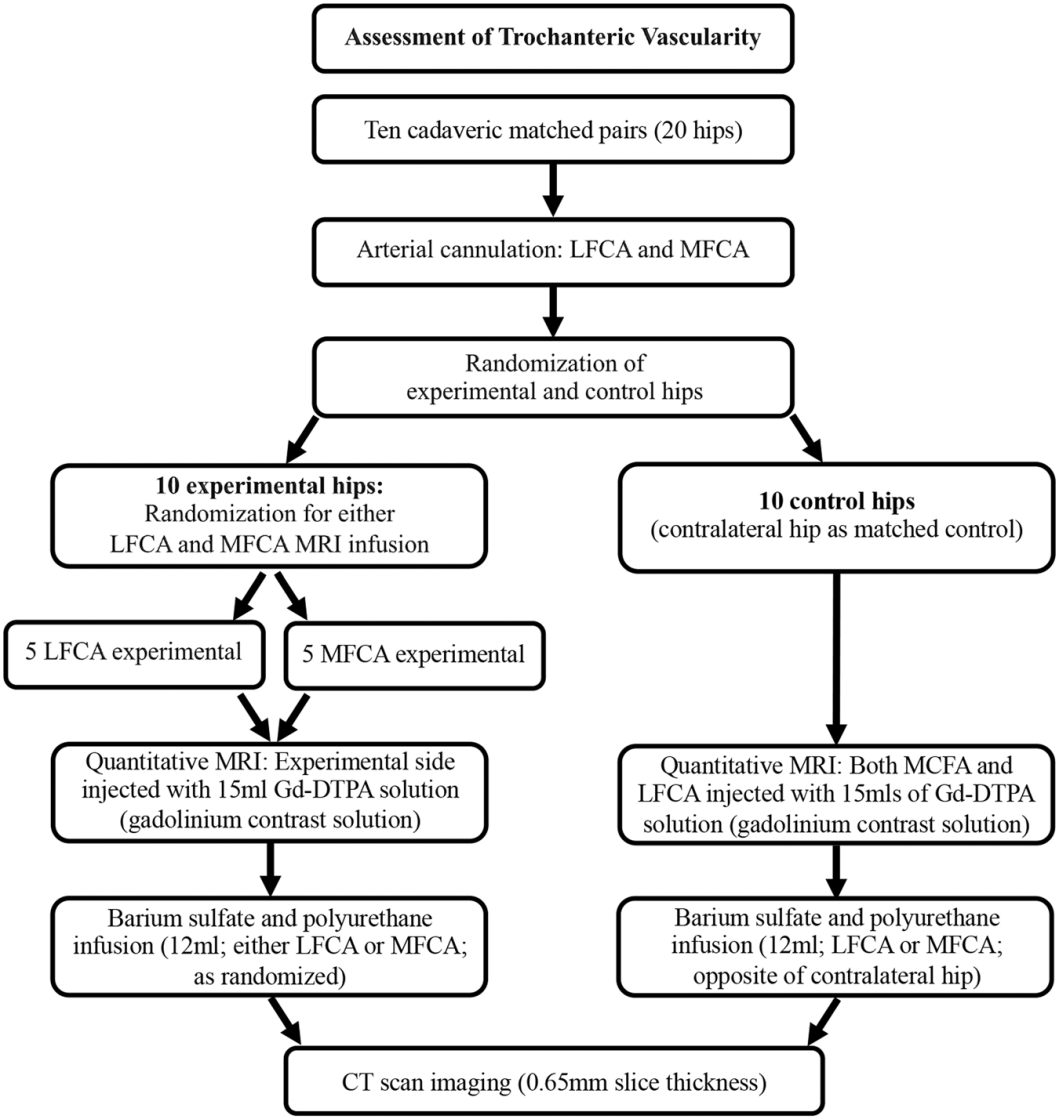
Flowchart of the research protocol.

**Figure 2:**
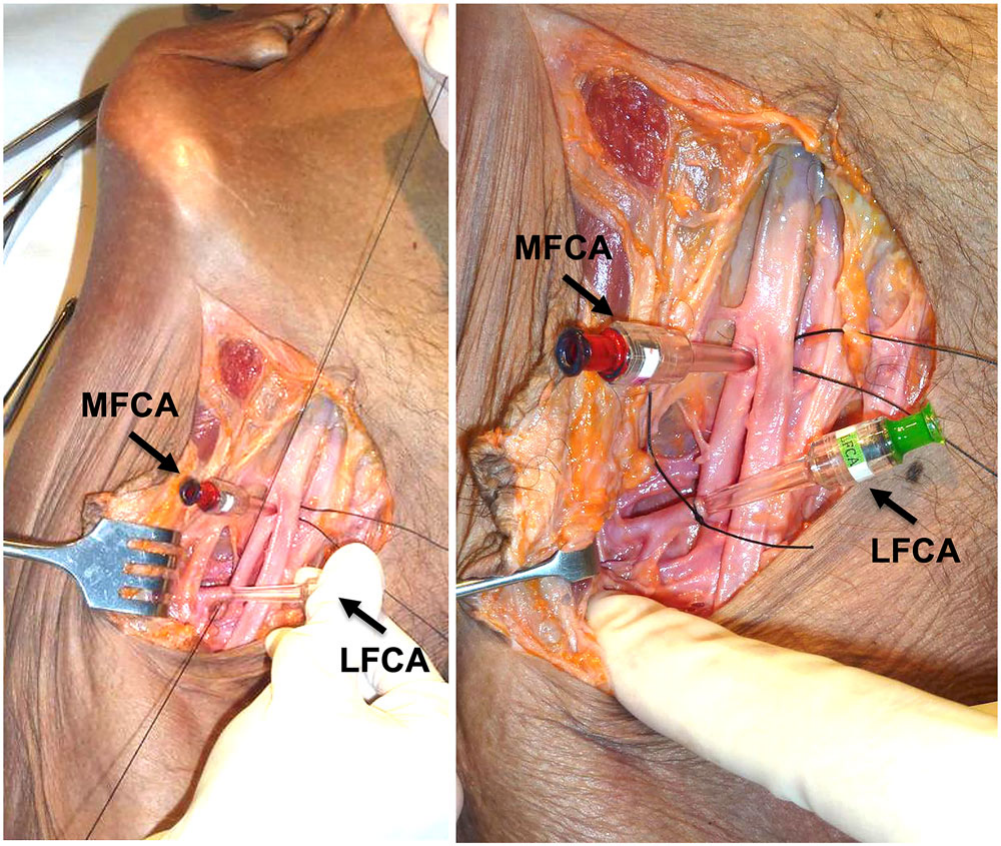
Images of the experimental setup illustrating the insertion of the vascular cannulas (model 30000; Medtronic) sutured securely into place using silk 2‐0 sutures (Ethicon Black Braided, Johnson & Johnson) for subsequent arterial infusion of MRI contrast agent. MRI, magnetic resonance imaging.

For each pelvic specimen, one hip was randomly assigned to serve as either LFCA‐experimental or MFCA‐experimental and the contralateral hip served as an internal matched control (Figure 1). Randomization was completed using Random Allocation Software (M. Saghaei, Isfahan, Iran). Bilateral anterior incisions were completed to expose and dissect the femoral artery. The origins of both the MFCA and LFCA were identified and then cannulated. Vascular cannulas (model 30000; Medtronic) were sutured securely into place using silk 2‐0 sutures (Ethicon Black Braided, Johnson & Johnson) for subsequent arterial infusion of MRI contrast agent (Figure 2).

Next, a previously established quantitative 3T MRI protocol was performed on the specimens consisting of 2 mm slice thickness and pre‐ and post‐infusion of MRI contrast solution using a ratio of 3:1 saline to gadolinium‐diethylenetriamine pentaacetic acid (Gd‐DPTA) [7, 19]. The control hip received contrast solution infusion by two separate syringes into both MFCA and LFCA (15 mL per artery with 30 mL total infusion). In the experimental hip, contrast solution was infused in either the LFCA or the MFCA based on randomization for a total infusion of 15 mL. Following the infusion of MRI contrast solution, a post‐contrast MRI scan was immediately performed.

To optimize quantitative MRI assessment, fat‐suppressed LAVA MRI sequences were utilized. This sequence minimizes the signal generated by bone marrow tissue and enhances the Gd‐DPTA imaging detail optimally for trabecular bone. A previously validated method for quantitative MRI analysis was utilized [5, 7, 19, 27].

### Quantitative MRI analysis

**Figure 3:**
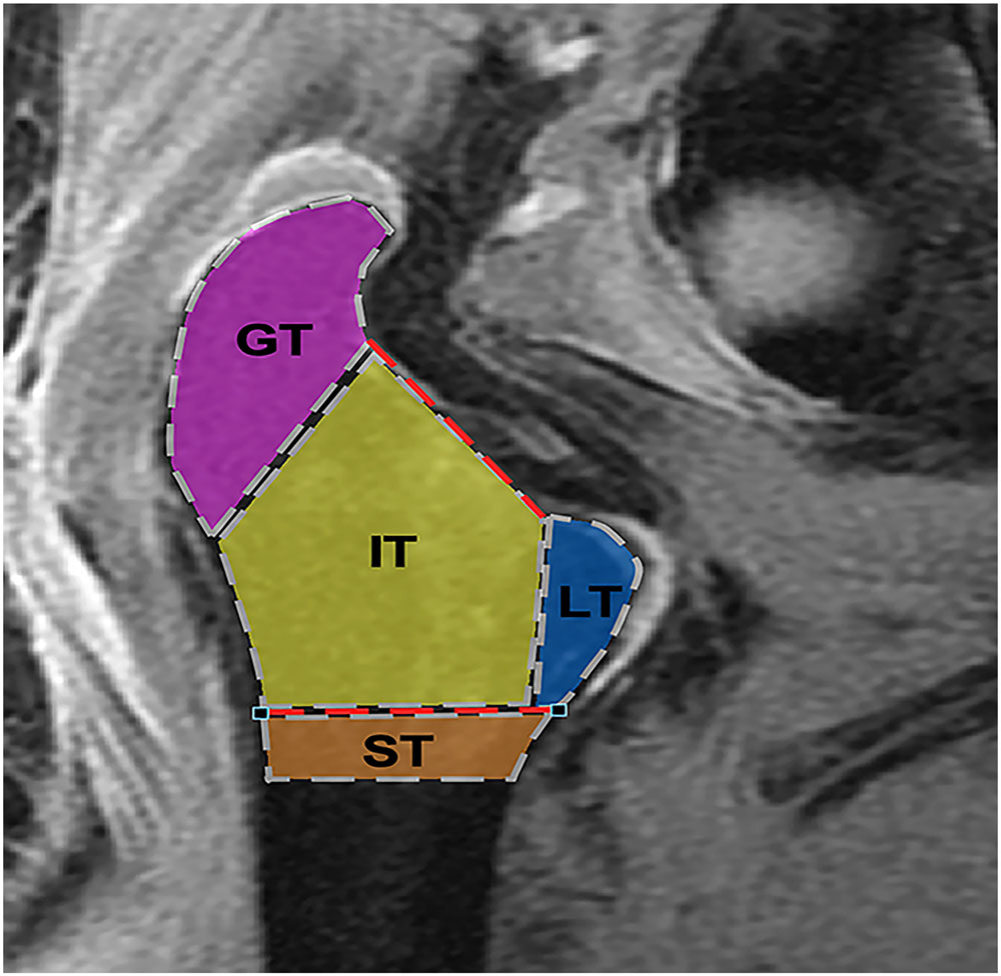
The trochanteric region was segmented into four regions of interest for zonal analysis including the following regions: (1) greater trochanter (GT), (2) intertrochanteric (IT), (3) lesser trochanter (LT) and (4) subtrochanteric (ST).

One study investigator (JPD) developed software for the quantitative MRI protocol using Interactive Data Language (IDL 6.4; Exelis), allowing region of interest (ROI) assessment along the coronal imaging plane. ROI delineation included the complete intraosseous trabecular area of the following regions: (i) greater trochanter (GT), (ii) intertrochanteric (IT), (iii) lesser trochanter (LT) and (iv) subtrochanteric (ST) region, and matching ROI areas were then delineated in the corresponding contralateral matched control hip to compare signal intensity within the same osseous regions (Figure 3). Each designated region was further divided into anterior and posterior subregions. One investigator performed all ROI measurements for the ROI analysis. Signal intensity was measured both before and after contrast solution infusion. A weighted average of signal intensity measurements and associated histograms were produced for both pre‐ and post‐contrast MRI images, which were normalized using a baseline of non‐enhancing muscle tissue. A single measurement of signal enhancement was determined for each ROI area for both control and experimental hips after obtaining weighted average of signal intensity per voxel for each study specimen. Normalized signal enhancement measurements from both the experimental and contralateral internal matched control hips were compared through corresponding ROI segments to assess the relative arterial contributions from the LFCA and MFCA.

### CT scan imaging

Following MRI, infusion with a polyurethane compound (Smooth‐On PMC‐780) mixed with barium sulfate (BaSO_4_) suspension at a 33% concentration (Liquid Polibar Plus 900203; Bracco Diagnostics Inc.) was performed of either the MFCA or LFCA based on randomization protocol described above. After polymerization at 20 h, thin‐slice computed tomography (CT) scan imaging was acquired (along the longitudinal axis at 0.65 mm slice thickness) with subsequent 2D‐ and 3D‐reformatting to assess arterial distribution, extraosseous and intraosseous course LFCA and MFCA, and assess arterial terminal branch locations and correlate findings with MRI.

### Statistical analysis

Statistical analysis was performed using SPSS software (version 1.0.0.1461; IBM). Data were tested for normal distribution using the Shapiro–Wilk test. Mann–Whitney *U* tests were used to compare quantitative MRI findings between regional arterial contributions, and a *p* value of <0.05 was considered statistically significant. The arterial contributions to the trochanteric regions are reported as mean and standard deviation and as median and interquartile range (IQR: 25%–75%) along with range.

### Source of funding

This investigation was funded through departmental research funding at the author's institution.

## RESULTS

**Figure 4:**
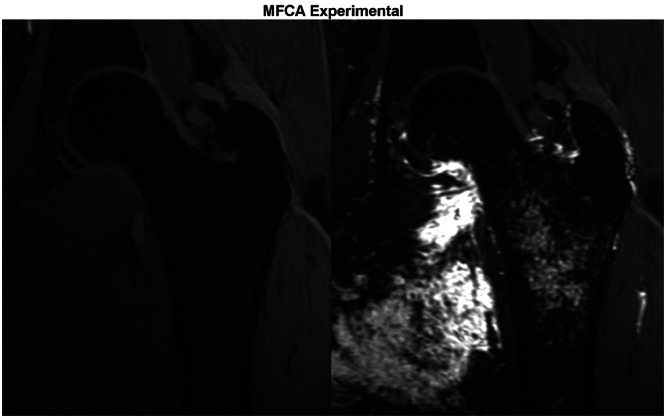
MRI, including pre‐contrast (left image) and post‐contrast (right image), demonstrating arterial contributions from the MFCA. MFCA, medial femoral circumflex artery; MRI, magnetic resonance imaging.

**Figure 5:**
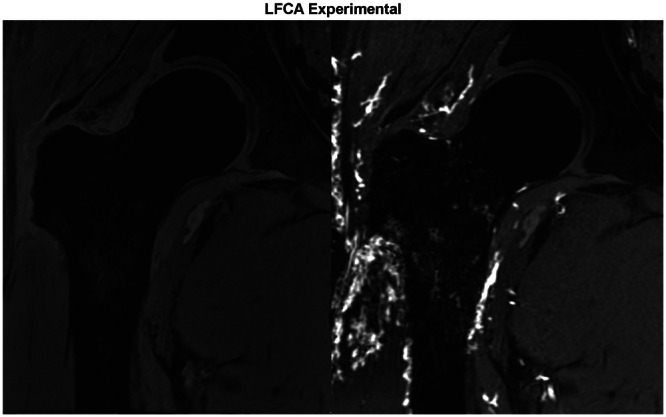
MRI, including pre‐contrast (left image) and post‐contrast (right image), demonstrating arterial contributions from the LFCA. LFCA, lateral femoral circumflex artery; MRI, magnetic resonance imaging.

**Figure 6:**
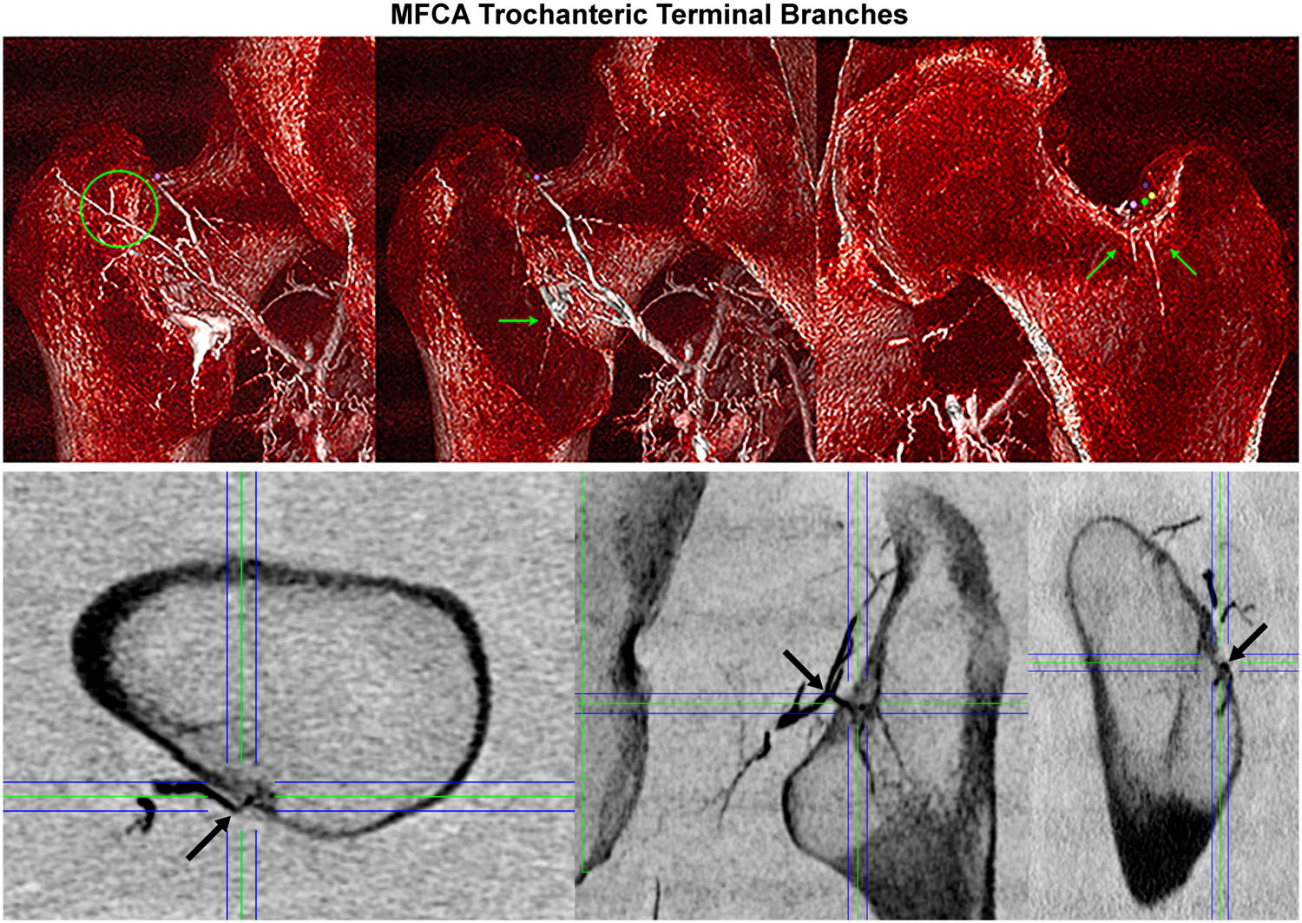
Subtraction‐CT (top images) and 2D‐CT orthogonal multiplanar reconstruction (MPR) imaging (bottom images) illustrating locations of terminal branches of the MFCA (as marked with arrows). 2D‐CT MPR (axial plane in bottom‐left image, coronal plane in bottom‐middle image and sagittal plane in bottom‐right image) illustrate a consistent terminal branch arising from the ascending branch of the MFCA and providing arterial contribution along the distal aspect of the intertrochanteric crest. 2D, two‐dimensional; CT, computed tomography; MFCA, medial femoral circumflex artery.

**Figure 7:**
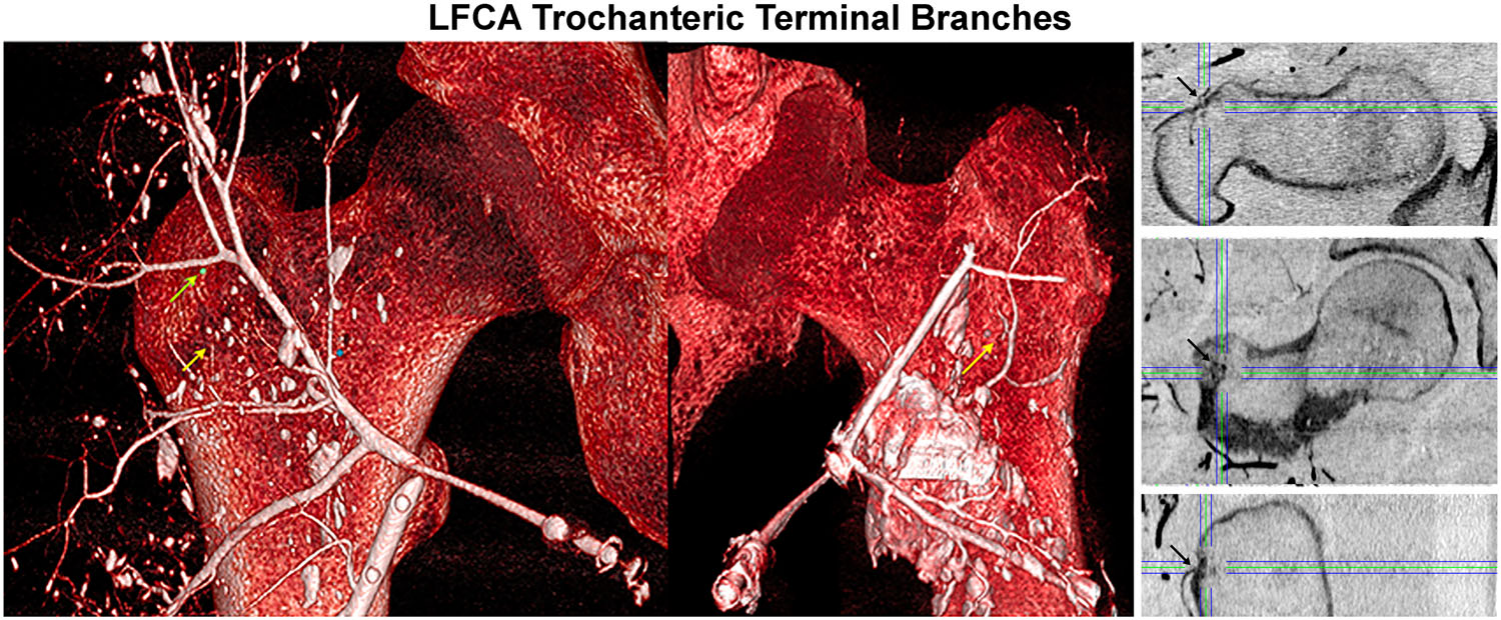
Subtraction‐CT (left images) and 2D‐CT orthogonal multiplanar reconstruction (MPR) imaging (right images) illustrating locations of terminal branches of the LFCA (as marked with arrows). 2D‐CT MPR (axial plane in right‐top image, coronal plane in right‐middle image and sagittal plane in right‐bottom image) illustrate a consistent terminal branch arising from the transverse branch of the LFCA and providing arterial contribution along the greater trochanter. 2D, two‐dimensional; CT, computed tomography; LFCA, lateral femoral circumflex artery.

The quantitative arterial contributions of the MFCA and LFCA are provided in Table 1. The MFCA provided the majority of arterial contributions to the entire trochanteric region (68.5% MFCA vs. 31.5% LFCA; SD: 10.7%, *p* < 0.001) (Figures 4 and 5). Over 70% of the blood supply to the ST, LT and IT regions arose from the MFCA. The blood supply to the GT was more balanced (52.5% from MFCA and 47.5% from LFCA; SD: 33.7%, *p* = 0.0853). Significant differences were found between the MFCA and LFCA arterial contributions in all trochanteric regions except for the greater trochanter (Table 1). CT assessment revealed multiple consistent trochanteric terminal branches providing osseous contributions from terminal branches of the MFCA and LFCA, as detailed in Table 2 and illustrated in Figures 6 and 7.

**Table 1:** Relative arterial contributions to the trochanteric region: Medial femoral circumflex artery (MFCA) versus lateral femoral circumflex artery (LFCA).

Region of perfusion	MFCA				LFCA				*p*
	Mean	SD	Median	Interquartile range (IQR)	Mean	SD	Median	IQR	
Full trochanteric region	**68.5%**	10.7%	68.9%	62.9%–71.9%	**31.5%**	10.7%	31.1%	28.1%–37.1%	**<0.001** *
Region analysis									
Subtrochanteric (ST)	**76.0%**	19.4%	75.0%	72.7%–86.8%	**24.0%**	19.4%	25.0%	13.2%–27.3%	**<0.001** *
Lesser trochanter (LT)	**71.4%**	37.1%	85.5%	63.3%–100.0%	**28.6%**	37.1%	14.5%	0.0%–36.7%	**0.035** *
Intertrochanteric (IT)	**74.0%**	25.2%	73.7%	68.2%–92.5%	**26.0%**	25.2%	26.3%	7.5%–31.8%	**0.004** *
Greater trochanter (GT)	**52.5%**	33.7%	65.7%	21.5%–74.6%	**47.5%**	33.7%	34.3%	25.4%–78.5%	0.853
Subregion analysis									
ST anterior	**80.8%**	21.2%	87.6%	65.7%–100.0%	**19.2%**	21.2%	12.4%	0.0%–35.3%	**<0.001** *
ST posterior	**71.1%**	35.1%	84.1%	50.5%–100.0%	**28.9%**	35.1%	15.9%	0.0%–49.5%	**0.029** *
LT anterior	**70.0%**	38.5%	88.7%	48.8%–100.0%	**30.0%**	38.5%	11.3%	0.0%–51.2%	**0.043** *
LT posterior	**72.8%**	38.5%	99.4%	54.2%–100.0%	**27.2%**	38.5%	0.6%	0.0%–45.8%	**0.019** *
IT anterior	**70.0%**	31.6%	73.8%	57.6%–98.7%	**30.0%**	31.6%	26.2%	1.3%–42.4%	**0.009** *
IT posterior	**78.0%**	29.3%	87.6%	73.7%–100.0%	**22.0%**	29.3%	12.4%	0.0%–26.3%	**<0.001** *
GT anterior	**50.3%**	36.1%	50.0%	22.9%–75.8%	**49.7%**	36.1%	50.0%	24.2%–77.1%	0.971
GT posterior	**54.8%**	36.7%	58.0%	25.0%–84.3%	**45.2%**	36.7%	42.0%	15.7%–75.0%	0.436
Full trochanteric anterior	**67.8%**	13.5%	68.7%	59.7%–77.1%	**32.2%**	13.5%	31.3%	22.9%–40.3%	**<0.001** *
Full trochanteric posterior	**69.2%**	14.3%	69.6%	59.6%–79.9%	**30.8%**	14.3%	30.4%	20.1%–40.4%	**<0.001** *

*Note*: Ranges are reported as IQR (25–75th percentiles).

Abbreviation: SD, standard deviation.

* Significant differences using the Mann–Whitney *U* test with significance set at *p* < 0.05.

**Table 2:** Findings from contrast‐enhanced CT imaging.

		Terminal branches			Max
Arterial source		Mean	SD	Min	
MFCA	Femoral neck (supero‐lateral aspect)	1.4	0.5	1	2
MFCA	Greater trochanter	1.7	0.5	1	2
MFCA	Intertrochanteric crest (distal aspect)	1.3	0.5	1	2
	Mean total	4.4			
LFCA	Femoral neck (anterior aspect)	1	0	1	1
LFCA	Inferior greater trochanter (anteriorly)	1.1	0.3	1	2
LFCA	Superior greater trochanter (anteriorly)	1.4	0.7	1	3
	Mean total	3.5			

Abbreviations: CT, computed tomography; LFCA, lateral femoral circumflex artery; MFCA, medial femoral circumflex artery; Max maximum; Min, minimum; SD, standard deviation.

## DISCUSSION

The MFCA was found to be the dominant arterial contribution to the trochanteric region, responsible for 68.5% of arterial contributions. However, in the GT region, MFCA accounted for 52.5% of the vascular contribution with the remainder from the LFCA. These findings suggest that if nutrient arterial supply from either the MFCA or LFCA is compromised by the fracture pattern or displacement, collateral blood supply may be provided by the remaining circumflex artery. However, compromise of the MFCA may have more impact on fracture healing compared to the LFCA due to its dominant arterial contribution.

A prior study by Najima et al. used 17 human cadavers (32 femurs) to examine the vascular anatomy of the GT after digastric versus trochanterotomy using either injection of latex lead oxide mixture for angiography, India ink and gelatin mixture, or a combination approach [23]. The authors found three major sources of GT blood supply: (1) the proximal soft tissues, including gluteus medius and minimus, supplied mainly by the internal iliac artery system; (2) the distal soft tissues, including vastus lateralis, supplied by the descending branches of the LFCA; (3) possible contribution from the transverse branch of the LFCA [23]. While our study primarily used quantitative MRI and focused on relative arterial contributions to trochanteric trabecular bone regions, our findings align with those from Najima et al., as we also found terminal branches from the LFCA provided substantial contributions to the GT (47.5% overall).

A study by Churchill et al. used a barium sulphate suspension to study the intraosseous vasculature of the greater trochanter in 16 human cadaveric femurs using microfocal radiography and histology. The common iliac artery was used to perform vascular infusion. The authors found several anastomoses between the GT nutrient vessels and adjacent cancellous bone. The authors also found that arterial vessels entered the GT from medial, lateral, and superior aspects, which aligns with our CT findings.

A study by Dewar et al. comparing the relative contributions of the MFCA and LFCA in the proximal femur, found the MFCA supplied 82% of the femoral head, a more significant contribution than that of the trochanteric region. The femoral neck received 67% of its vascular contributions from the MFCA and 33% from the LFCA, which is similar to that of the trochanteric region [7].

Fractures along the trochanteric region of the proximal femur are common. A cross‐sectional study by Diaz and Navas of 428 patients over age 65 admitted for treatment of trochanteric or femoral neck fractures found that 51.4% had femoral neck fractures and 48.6% trochanteric fractures [8]. A study by Mattisson et al. from the Swedish Fracture Registry from January 2014 to December 2016 found that among 10,548 fractures with ICD‐10 codes denoting trochanteric or subtrochanteric fractures, 78% were trochanteric, and 22% were subtrochanteric [22]. While modern fixation constructs can result in higher rates of union, nonunion remains a clinical concern due to a high overall prevalence of trochanteric fractures [4].

Care should also be taken to protect the nutrient arterial supply, as with all fracture surgery. Surgical approaches may result in damage to the blood supply of the trochanteric region. A study by Schottel et al. assessed the impact on vascularity of piriformis fossa and trochanteric entry points used during antegrade femoral nailing in a cadaveric model using quantitative MRI [26]. While the authors found no significant differences between groups in arterial contribution to the femoral head, femoral head‐neck junction or femoral neck vascularization, in 40% of piriformis fossa group specimens, arterial entry points were within 1 mm of the deep branch of the MFCA. This may have impacted trochanteric vascularization which was not assessed in the study by Schottel et al. As the authors noted, caution is warranted against multiple errant starting point attempts and meticulous soft tissue protection is important [26].

A retrospective cohort study by Berk et al. on 225 patients aged >70 years with a diagnosis of trochanteric femur fractures found an overall nonunion rate of 9%. Nonunion was significantly more common with AO/OTA Type 31A3 fractures (23.3%) as compared with Type 31A2 (6.9%) or AO/OTA 31A1 (3.2%) (*p* < 0.001). AO/OTA Type 31A3 fractures involve the IT region. Damage to MFCA and LFCA terminal branches to the IT region may result from the injury or during surgical repair. As the patients in the study by Berk et al. were treated using femoral intramedullary nails (Gamma nails), it is unlikely that the deep branch of the MFCA was compromised during nail insertion. Therefore, the injury pattern associated with AO/OTA Type 31A3 may be associated with increased arterial compromise to nutrient branches of the LFCA and MFCA leading to higher nonunion rates observed in the study by Berk et al. Multiple branches from the MFCA and LFCA enter the trochanteric region along the IT region as demonstrated in the CT findings presented in this study.

Surgical risk factors also affect nonunion rates following ORIF of trochanteric fractures and include mail reduction, inadequate debridement of the fracture site, and fixation constructs with inadequate stability [1]. Open reduction and cerclage were not associated with nonunion, suggesting that these techniques spare enough vascularity to be compatible with fracture healing [4]. It is important to consider the trochanteric blood supply and impact of surgical approaches on the hip during treatment of fractures.

This quantitative MRI analysis of the femoral trochanteric region arterial contributions confirmed our hypothesis that the MFCA provides the dominant arterial supply to the trochanteric region, emphasizing its overall importance to hip vascularity. However, the LFCA provides a nearly equal contribution to GT vascularity, highlighting a dual arterial contribution to this anatomic region. Hartog et al. retrospectively reviewed 1280 femoral digital subtraction arteriograms and found that arterial perfusion to the greater trochanter was impaired following anterior surgical approaches that involve ligation of the LFCA [15]. This is consistent with the substantial role in GT arterial contributions from the LFCA found in the present study.

The greater trochanter plays a significant role in surgical approaches, as it can be osteotomized to improve surgical exposure to hip and femur. Nonunion following osteotomy or fracture is of concern especially in the setting of previous hip surgery or total hip arthroplasty, with rates as high as 6% [6, 24]. Consideration of prior surgical approaches is crucial in maintaining vascularity during osteotomies or surgical fixation of fractures, as prior anterior approaches may reduce LFCA arterial contributions, and prior posterior approaches can negatively impact MFCA arterial contributions.

This study does have limitations. Contrast‐enhanced MRI was performed through infusion of the retinacular arterial system (MFCA and LFCA) based on prior research evidence of the retinacular system providing the dominant arterial supply to the femoral head and neck [7, 10]. Interosseous or foveolar arterial contributions were not assessed. This research investigation did not specifically assess for reliability and reproducibility associated with the MRI ROI analysis which would have been beneficial. However, MRI findings in this study were further supported by the CT findings with respect to terminal branches from both the MFCA and LFCA. Additionally, gadolinium MRI contrast solution was infused manually without the use of an infusion pump which may have introduced variability. However, one study investigator performed all infusions at the time of MRI in an attempt to minimize the potential for pressure‐related variation during MRI contrast solution infusions.

This study has several strengths. It is the first quantitative study assessing the arterial contributions to the trochanteric regions of the femur. Additionally, CT imaging was performed, which allowed the MRI results to be further qualitatively assessed. The CT imaging also revealed multiple consistent MFCA and LFCA terminal branches providing osseous perfusion to the trochanteric region, which adds to the literature.

## CONCLUSIONS

Quantitative MRI results demonstrated that the MFCA provided the dominant trochanteric arterial supply. This further highlights MFCA's importance to overall hip perfusion. LFCA's contribution to trochanteric vascularity is smaller but still contributes to the trochanteric region, especially to the GT region. Expanded knowledge of the native arterial supply to the trochanteric region can have further implications for optimizing surgical approaches for the treatment of trochanteric fractures and bone healing in trochanteric fracture patterns, trochanteric nonunions and trochanteric osteotomies.

## AUTHOR CONTRIBUTIONS

**Craig E. Klinger**: Conceptualization; data curation; formal analysis; investigation; methodology; project administration; writing—original draft; writing—review and editing. **Barak Altintas**: Investigation; project administration; methodology; writing—original draft; writing—review and editing. **Kathryn A. Barth**: Methodology; supervision; visualization; writing—original draft; writing—review and editing. **Kenneth M. Lin**: Methodology; supervision; visualization; writing—original draft; writing—review and editing. **David C. Dewar**: Conceptualization; data curation; methodology; visualization; writing—original draft; writing—review and editing. **Lionel E. Lazaro**: Conceptualization; data curation; methodology; visualization; writing—original draft; writing—review and editing. **Jonathan P. Dyke**: Methodology; resources; software; validation; writing—original draft; writing—review and editing. **David S. Wellman**: Conceptualization; methodology; supervision; writing—original draft; writing—review and editing. **David L. Helfet**: Conceptualization; methodology; supervision; writing—original draft; writing—review and editing.

## CONFLICT OF INTEREST STATEMENT

David L. Helfet reports ownership interest in Healthpoint Capital, LP and Advisory Board membership in Diamond Orthopaedics, and OHK Medical Devices, Inc., outside of the scope of the submitted work. David S. Wellman reports personal fees from DePuy Johnson and Johnson, and OrthoDevelopment outside of the scope of the submitted work. The remaining authors declare no conflict of interest.

## ETHICS STATEMENT

Approval for this cadaveric vascularity study was obtained from our institutional review board prior to initiation of this investigation. Consent to participate was not applicable due to the nature of this study.

## Data Availability

The data sets used and/or analyzed during the current study are available from the corresponding author on reasonable request.
